# Effect of Fixed and Removable Functional Therapy on Mandibular Anterior Bone Structures: A Fractal Analysis Study

**DOI:** 10.3390/diagnostics14161713

**Published:** 2024-08-07

**Authors:** Orhan Cicek, Deniz Arslan

**Affiliations:** Department of Orthodontics, Faculty of Dentistry, Zonguldak Bulent Ecevit University, Zonguldak 67600, Türkiye; d.arslan@beun.edu.tr

**Keywords:** functional therapy, fractal analysis, trabecular bone, lateral cephalometric radiograph

## Abstract

(1) Background and aim: The effects of functional therapies on dentoalveolar and skeletal structures have been investigated in orthodontics for many years. The aim of this retrospective study was to evaluate the changes caused by fixed and removable functional therapy in the mandibular anterior trabecular structures using fractal dimension (FD) analysis. (2) Methods: A total of 60 patients with skeletal and dental class II malocclusion were included in the study and three groups were formed: the untreated control group (CG), the Forsus fatigue-resistant device group (FFRDG), and the Monoblock group (MBG). Bone areas of interest determined in the buccoapical of the mandibular incisors and the symphysis in the lateral cephalometric radiographs taken before (T0) and after (T1) functional therapy were evaluated using FD analysis. The relationship between the FD and IMPA (Incisor Mandibular Plane Angle) angles was evaluated. Parametric and nonparametric tests were used in statistical analysis according to normality distribution. The statistical significance level was determined as *p* < 0.05. (3) Results: There was no statistically significant difference between the FD values of all groups at T0 (*p* > 0.05). At T1, buccoapical FD values were significantly lower in FFRDG and MBG compared to the control group (*p* < 0.05), while symphyseal FD values were not found to be significant (*p* > 0.05). The IMPA angle was significantly lower in the FFRDG and MBG than in the control group at T0, while it was higher at T1 (*p* < 0.05). While a significant negative correlation was observed between the IMPA angle and buccoapical FD values in both FFRDG and MBG (*p* < 0.05), it was not observed with the symphysis FD values (*p* > 0.05). (4) Conclusions: Trabecular changes caused by functional therapy in the mandibular anterior bone can be evaluated on lateral cephalometric radiographs with FD analysis. It was concluded that orthodontists should ensure controlled changes in the IMPA angle during functional therapy, especially for the decreases in FDs seen in the buccoapical alveolar region due to the forward movement of the mandibular incisors.

## 1. Introduction

The term ‘fractal’ refers to structures with irregular dimensions that cannot be defined by classical geometry and was first used to describe complex structures in the 1960s and 1970s [1]. Unlike the mathematical concept of fractals, biological fractals express the complexity of structures that do not repeat at different scales or within themselves. Biological fractals include structures such as bronchial system branches, the artery–vein complex, and trabecular bone [2]. Numerous scientific fields, including dentistry, have made extensive use of the fractal dimension (FD) analysis method, which makes it possible to express complex structures by turning them into numerical data [3]. Previous studies have shown that FD analysis, which can be applied to dental radiographs, can serve as a therapy guide by reflecting mineral changes in the trabecular bone structure [4,5,6].

Class II malocclusions are one of the most common orthodontic anomalies, affecting roughly one-third of the population [7]. Skeletal class II malocclusions, which are sagittal direction anomalies induced by hereditary or environmental etiological reasons, can originate from maxillary excess, mandibular deficiency, or both [8]. In such cases, diagnosing the jaw where the problem originated is crucial in the treatment plan. Mandibular growth can be stimulated in its developmental deficiency using fixed or removable functional appliances, depending on the growth and development period, age, and severity of malocclusion [9].

Functional appliances are well known for having both dental and skeletal effects. These appliances have been found to induce anterior movement of the mandibular base and dental arch while also causing posterior movement by transmitting distal forces to the maxillary dentoalveolar structures [10]. During these tooth movements, which are caused by the direct or indirect effect of functional forces on the jaws, resorption-apposition occurs in the trabecular bone structure [11]. It has been shown that the microstructure alterations that occur in trabeculation also affect bone morphology [12]. When reviewing the literature, it is clear that many studies use FD analysis to analyze the micro changes that occur in the jaw bones during orthodontic treatment [4,13,14,15].

There are previous studies investigating the changes caused by functional therapy in mandibular bone trabeculations using FD analysis with dental panoramic radiographs. Gümüş et al. [15] found that functional orthopedic therapy with Twinblock or Monoblock appliances significantly decreased FD values in the mandibular corpus for skeletal class II malocclusion. In contrast, Amuk et al. [14] reported that FD values in the mandibular angulus region increased during fixed orthodontic treatment after using the Herbst appliance. The structure of trabeculae is determined by porosity, thickness and anisotropy [16], and as the FD values obtained with FD analysis increase, which defines the complexity of a structure by measuring similarities within it, the complexity of the structure increases [17,18]. That is, a high FD value indicates that the structure examined is denser and more complex. Furthermore, trabecular losses during orthodontic treatment occur due to increased regional pressure caused by mechanical overloading [18]. This results in lower FD values, which means decreased density and complexity. Thus, FD analysis has been proposed as an objective diagnostic tool in both medicine and dentistry, as trabecular structures can be evaluated using FD analysis on radiographs with high-quality resolution [18,19].

Examining the orthodontic literature, we find a large number of studies that use FD analysis on panoramic radiographs to investigate the modifications brought about by functional orthopedic therapy in structures such as bones and condyles [13,14,20]. However, no study has been found that evaluates the changes occurring in the mandibular anterior bone region after the use of functional appliances with FD analysis on lateral cephalometric radiographs. Therefore, this study aimed to evaluate the changes in the trabeculation of the mandibular anterior alveolar bone—which is affected by the movement of the lower incisors—and mandibular symphysis after fixed and removable functional therapies using the FD analysis method on lateral cephalometric radiographs.

The first null hypothesis of this study is that there is no significant difference between the FD values of the groups after functional therapy. The second null hypothesis of this study is that there is no significant correlation between post-treatment IMPA (Incisor Mandibular Plane Angle) and FD values.

## 2. Materials and Methods

### 2.1. Study Design and Ethical Approval

This retrospective single-center study was conducted by analyzing the lateral cephalometric radiographs in clinical archive records. Ethics committee approval was obtained from Zonguldak Bülent Ecevit University Non-Interventional Clinical Research Ethics Committee (Date: 8 May 2024 and Decision No: 2024/09-6).

### 2.2. Sample Size Calculation and Groups

The sample size of this study was performed with the G*Power (version 3.1.9.7; Franz Faul, Universität Kiel, Kiel, Germany) program. Since there was no similar previous study, the effect size required for the sample size calculation of this study was obtained using the mean and standard deviation values of the IMPA angle in the groups at T1. Accordingly, considering the effect size of 0.5198432 obtained from the mean and standard deviations and the 5% α error probability, the real power of this study was calculated as 90% (non-centrality parameter λ = 13.7820846 and critical F = 3.1907273) when there were at least 51 total samples (17 samples per group). Therefore, to further increase the power of the study, 20 patients in each group, with a total of 60 patients, were included by scanning the clinical archive records of the Zonguldak Bülent Ecevit University Orthodontics Department. The patients were divided into three groups: a control group (CG, n = 20) who did not receive orthodontic treatment, a group treated with the Forsus (Forsus fatigue-resistant device)(3M, Monrovia, USA) fixed functional device group (FFRDG, n = 20), and a group treated with the Monoblock removable functional appliance (MBG, n = 20).

### 2.3. Inclusion and Exclusion Criteria

The inclusion criteria for the FFRDG and MBG study groups were defined as follows:

- Skeletal and dental class II malocclusion;
- No history of trauma;
- No previous orthodontic therapy;
- Completed functional orthodontic therapy;
- No systemic bone disease;
- For MBG, patients whose growth development is in the peak period (MP3 capping);
- For FFRDG, patients whose growth development is in the post-peak period (MP3 union);
- Good-quality high-resolution cephalometric radiographs.

The control group patients were selected from systemically healthy patients with skeletal and dental class II malocclusion and no previous orthodontic treatment. Patients who did not meet any of the inclusion criteria in all groups were excluded from the study. The growth and development periods of the study group patients were determined using hand–wrist radiographs.

### 2.4. Interventions and Cephalometric Measurements

Patients in the FFRDG were treated using a Forsus fatigue-resistant device (3M, Monrovia, USA) with 0.022 × 0.028-inch slot preadjusted MBT metal brackets. A 0.019 × 0.025-inch heat-activated rectangular nickel–titanium (NiTi) archwire and a 0.019 × 0.025-inch stainless steel rectangular archwire (American Orthodontics, Shehboygan, WI, USA) were placed after the placement of 0.012-inch, 0.014-inch, and 0.016-inch round heat-activated NiTi archwires and 0.016-inch round stainless steel archwire, respectively [21]. After leveling and alignment were completed, the Forsus fixed functional device was placed and kept in the mouth for 4.9 ± 1.1 months until the fixed functional therapy was completed. All patients in the removable functional therapy group were treated in a single stage with a Monoblock appliance, which was prepared with a maximum activation of 7 mm until the growth and development period was completed [22]. While the appliances were used without the need for patient cooperation in FFRDG, they were used for 16–18 h per day, except for eating and brushing teeth, until growth and development were completed in MBG. The endpoint for FFRDG is to have normal overjet and class I molar/canine relationship after therapy. The endpoint for MBG is to have passed the peak period and have normal overjet and class I molar/canine relationship. T1 period for FFRDG is after completion of fixed functional appliance therapy, which is placed 1 session after the 0.019 × 0.025-inch rectangular stainless steel archwire is placed in the fixed preadjusted MBT appliance. T1 period for MBG is immediately after completion of removable functional appliance therapy applied directly without prior leveling.

Measurements were made on lateral cephalometric radiographs taken with a cephalometric X-ray device (Veraviewepocs 2D, J Morita Mfg. Corp., Kyoto, Japan) before functional therapy (T0) and after functional therapy (T1) in the areas of interest. In order to ensure standardization while taking cephalometric radiographs, the head position was fixed with cephalostats and the Frankfurt Horizontal Plane was adjusted to be parallel to the ground. Angular measurements of all patients were made using radiographs obtained from the same cephalometric X-ray machine. IMPA (Incisor Mandibular Plane Angle) angles, which are the angle between the long axis of the lower incisor and the mandibular plane (Gonion–Menton line), were measured on these radiographs using the NemoCeph digital analysis program (Nemotec, 2006, Madrid, Spain).

### 2.5. Fractal Analysis

**Figure 1:**
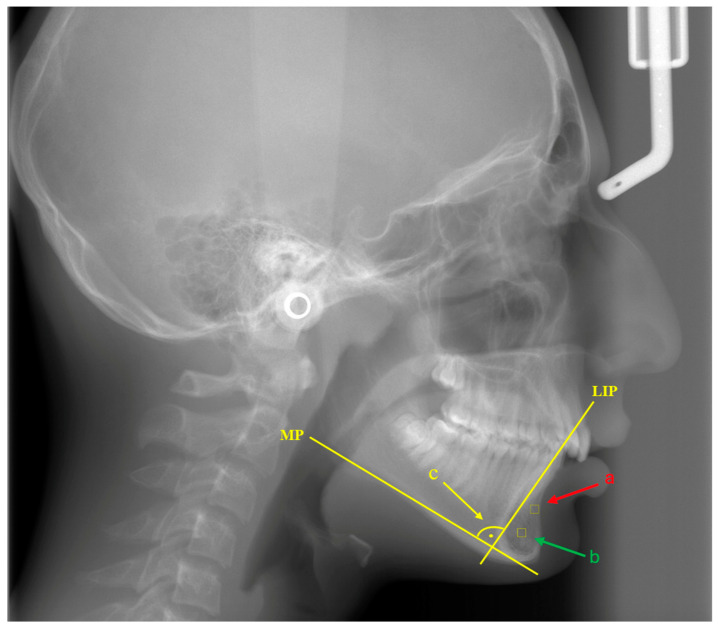
MP: mandibular plane. LIP: lower incisor long axis plane. a: Buccoapical area of the mandibular incisors. b: Mandibular symphysis area. c: IMPA angle.

Fractal dimension analysis was performed on lateral cephalometric radiographs using ImageJ (version 1.53), a JAVA-based image processing software from the National Institute of Health. The procedures required for FD analysis were performed on the same computer and by the same investigator using the box-counting method developed by White and Rudolph [23]. In the lateral cephalometric radiographs of all patients, areas of interest were identified from the buccoapical region of the mandibular incisors and the mandibular symphysis in the size range of 30 × 30 pixels. Attention was taken to ensure that there was no pathology, tooth root, or lamina dura in the areas of interest [4] (see Figure 1).

**Figure 2:**
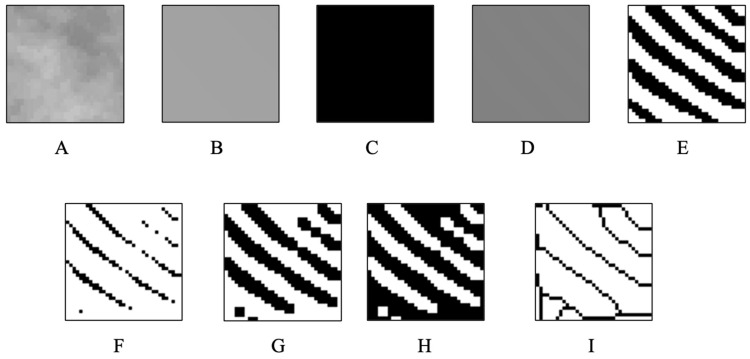
(**A**) Selected area from the original cephalometric radiograph, (**B**) Gaussian filtered image, (**C**) subtraction process, (**D**) 128 gray value added image, (**E**) binary, (**F**) erosion, (**G**) dilatation, (**H**) inversion of the processed image, (**I**) skeletonization.

After the selected area was copied and saved in 8-bit format, a ‘Gaussian Blur’ filter (sigma = 35 pixels) was applied to the copied image in order to eliminate factors that create imbalance in brightness, such as soft tissue. The resulting image was converted to ‘Binary’ format by subtracting the original image with ‘Subtraction’ process and adding 128 gray values. In order to remove the noise in the image, ‘Erosion’ and ‘Dilatation’ operations were applied, respectively, and then the ‘Invert’ option was applied. ‘Skeletonize’ was applied to the image to reveal the skeletal structure in the bone trabeculae. FD analysis with box-counting method was applied to the inverted image [4]. As a result of these process steps, a quantitative arbitrary value was formed and taken into account as the FD value. A decrease in FD values indicates a decrease in trabecular complexity and density, or vice versa. (see Figure 2).

### 2.6. Statistical Analysis

Statistical analysis was performed using SPSS 26 (Statistical Package for Social Sciences, IBM Co., NY, USA). Kolmogorov–Smirnov test was applied for normality distribution. For dependent data, paired sample *t*-test was used for normally distributed data, and Wilcoxon test was used for non-normally distributed data. For pairwise comparisons in independent groups, Student’s *t*-test was used for normally distributed data, while Mann–Whitney U test was used for non-normally distributed data. Intra-observer reliability was assessed using Cronbach’s α and two-way random effect intra-class correlation coefficients in all measurements of 20 randomly selected patients. The relationship between FD values and IMPA changes was evaluated using Spearman’s rho correlation coefficient. Statistical significance level was determined as *p* < 0.05.

## 3. Results

A high intra-observer reliability coefficient between 0.93 and 0.96 was found among repeated measurements made at two-week intervals.

Data regarding the age, gender, and therapy duration of the patients included in the study are given in Table 1.

In the mandibular buccoapical alveolar region:

At T0, the FD values of the CG, FFRDG, and MBG groups were found to be 1.745 ± 0.001, 1.743 ± 0.001, and 1.746 ± 0.001, respectively. There was no statistically significant difference between the FD values of the groups (*p* > 0.05). At T1, the FD values of the FFRDG and MBG groups were found to be 1.738 ± 0.011 and 1.736 ± 0.012, respectively. FD values of the FFRDG and MBG groups were significantly lower than the CG group (*p* < 0.05). There was no significant difference between the FD values of the FFRDG and MBG groups (*p* > 0.05). While there was a significant decrease in the FD values of the FFRDG and MBG groups at T1 compared to T0 (*p* < 0.05), no significant difference was observed between the T1/T0 change amounts (*p* > 0.05).

In the mandibular symphysis region:

At T0, the FD values of the CG, FFRDG, and MBG groups were found to be 1.745 ± 0.001, 1.744 ± 0.001, and 1.745 ± 0.001, respectively. At T1, the FD values of the FFRDG and MBG groups were found to be 1.743 ± 0.001 and 1.742 ± 0.01, respectively. In all groups, no significant differences were found in both intra- and inter-group comparisons (*p* > 0.05).

The results of the statistical analysis of intra- and inter-group FD values are presented in Table 2.

IMPA angle:

At T0, the mean IMPA values of the CG, FFRDG, and MBG groups were 93.9 ± 3.8, 90.8 ± 3.9, and 90.3 ± 3.0, respectively. While the IMPA angles of the FFRDG and MBG groups were significantly lower than the CG group (*p* < 0.05), no significant difference was found between them (*p* > 0.05).

At T1, the mean IMPA angles of the FFRDG and MBG groups were 97.5 ± 4.0 and 98.5 ± 4.5, respectively. While the IMPA angles of the FFRDG and MBG groups were significantly higher than the CG group (*p* < 0.05), no significant difference was found between them (*p* > 0.05). However, a significant increase was observed in the IMPA angles of the FFRDG and MBG groups at T1 compared to T0 (*p* < 0.05). Additionally, the amount of T1/T0 change was higher in the MBG group compared to FFRDG (*p* < 0.05).

The results of the statistical analysis of intra- and inter-group IMPA angles are presented in Table 3.

According to Spearman’s rho correlation coefficients:

In FFRDG, a statistically significant negative correlation was found between IMPA angles and buccoapical FD values (*p* < 0.05). However, no significant correlation was found between IMPA and symphysis FD values (*p* > 0.05). Similarly, no significant relationship was found between buccoapical and symphysis FD values (*p* > 0.05).

In MBG, a statistically significant negative correlation was found between IMPA angles and buccoapical FD values (*p* < 0.05). However, no significant correlation was found between IMPA and symphysis FD values (*p* > 0.05). Similarly, no significant relationship was found between buccoapical and symphysis FD values (*p* > 0.05). Correlation statistical results in groups are shown in Table 4.

## 4. Discussion

In this study, the changes caused by functional therapy with fixed and removable appliances in the mandibular anterior trabecular bone were evaluated using the FD analysis method using lateral cephalometric radiographs. After functional therapy, buccoapical FD values of FFRDG and MBG were found to be significantly lower than before therapy. In contrast, the decreases in the FD values of the symphysis region were not found to be significant. It was observed that IMPA values of the study groups, which were significantly lower compared to CG in the T0 period, increased significantly with functional therapy. However, a significant negative correlation was found between the decrease in buccoapical FD values and the increased IMPA angles. Based on these results, the first and second null hypotheses of this study were rejected.

Micro-computed tomography is considered the gold standard in the evaluation of bone structure, but it is not suitable for clinical diagnosis due to the need for relevant tissue sections and high radiation exposure [24]. Similarly, although cone beam-computed tomography helps in the three-dimensional evaluation of bone tissue, it has not taken its place among routine clinical diagnostic tools [25]. For this reason, in this study, lateral cephalometric radiographs, which are among the routine diagnostic records taken from orthodontic patients, were used to quantitatively evaluate the bone structure with FD analysis.

Fractal dimension analysis allows changes in bone density to be expressed as numerical data through measurements made on dental radiographs without requiring any invasive procedures [26]. In today’s dentistry, FD analysis has found wide use in many areas, such as monitoring the healing period after endodontic treatment [27], evaluating bone tissue around implants [28], examining the changes in the condyle structure of pediatric patients with bruxism [29], evaluating the degree of alveolar bone destruction in patients with periodontitis [30], examining the microstructure of composites used in restorative dentistry [31], and evaluating the effects of orthodontic treatment on alveolar bone structure [32]. In this study, FD analysis was used to evaluate trabecular mineralization changes in the mandibular anterior bone structures before and after treatment of patients treated with removable and fixed functional appliances. In this respect, this presented study is the first to investigate the effect of functional therapy on the mandibular anterior bone structures by FD analysis on lateral cephalometric radiographs.

It has been reported that FD values in normal or healthy trabecular bone in the jaws have been measured as approximately 1.5 on periapical radiographs [33] and that FD values ranging from 1.05 to 1.74 have been obtained in other studies [1,34,35]. It is thought that these differences in FD values may be due to variations in image parameters or different ROI sizes [34]. However, this presented study is the first comprehensive study to perform FD analysis on lateral cephalometric radiographs, and the obtained FD values are consistent with the literature.

Functional appliances aim to correct the skeletal and occlusal sagittal relationship by stimulating the growth of the mandible, which is positioned further back than its normal position [36]. In functional orthodontic therapy, it is inevitable that dental movements will occur as well as skeletal effects. This can be explained by the transfer of muscle forces generated by positioning the mandible more forward to the periosteum and from there to the bone tissue via dentoalveolar structures [37]. In the study by Bilgiç et al. [38], comparing the skeletal and dental effects of functional appliances, it was reported that Activator and Forsus appliances produced similar levels of skeletal correction and that both appliances had proclining and intrusive effects on the mandibular incisors.

In this presented study, the effect of tooth movements on the mandibular anterior teeth caused by functional therapy with Forsus and Monoblock appliances on the trabecular bone structure in the relevant region was evaluated. It was observed that there was a significant increase in IMPA angles of the patients treated with Forsus and Monoblock appliances, while there was a significant decrease in FD values of the mandibular buccoapical alveolar region. Additionally, a significant negative correlation was found between the decrease in buccoapical FD values and the increased IMPA angles.

There are areas of compression and tension in the periodontal ligament since orthodontic forces are transferred to the tooth. This situation initiates a series of resorption and apposition processes in the periodontal tissues adjacent to the tooth roots. Orthodontic tooth movement occurs during these changes in surrounding alveolar bone mineralization [11]. Wagle et al. [39] conducted experimental research on 24 rats with forces ranging from 0.1 to 0.5 N in order to examine the changes caused by orthodontic tooth movement at the periodontal ligament (PDL) and bone interface. They concluded that the applied orthodontic force caused an increase in the PDL–bone interface FD values of the relevant tooth and that this increase was directly proportional to the amount of applied force. Similarly, Cicek et al. [4] reported that they observed an increase in FD values in the compressed alveolar bone in the direction of movement after orthodontic space closure in congenital maxillary lateral incisor missing.

In this presented study, decreases in FD values were observed in the functional therapy groups due to the remodeling of the buccoapical alveolar bone, which mostly occurs by resorption due to the forward movement of the mandibular incisors. In the studies of Wagle et al. [39] and Cicek et al. [4], the increase in FD values in the direction of movement is due to the fact that there is a real compression area along the dental arch during tooth movement. In our study, on the contrary, the mandibular incisors were directed to move out of the dental arch with functional therapy. This resulted in a decrease in the trabeculation of the alveolus in the relevant region and therefore a decrease in FD values. The statistically significant negative correlation between the IMPA angle and the FD of the buccoapical alveolar bone confirms this finding. Therefore, in our study, it was emphasized to examine the trabeculation of the bone in the region with FD analysis and to carefully evaluate the IMPA angle before treatment in order to realize the desired trabecular remodeling processes due to the forward movement of the mandibular incisors during functional therapy.

It is seen that many studies in the orthodontic literature use FD analysis to evaluate the changes in bone tissue with orthodontic treatment [14,15,32,40,41]. Amuk et al. [14] stated that no change was observed in mandibular angulus FD values during the use of the Herbst appliance, but there was an increase in FDs in orthodontic treatment after Herbst. In another study, Gümüş et al. [15] found that functional therapy with Twinblock or Monoblock appliances did not show a significant difference in mandibular condyle FD values. In this study, it was observed that buccoapical FD values at T1 were significantly lower in the study groups compared to the control group, but there was no significant change in the symphysis region. Additionally, there was no significant difference between the T0/T1 change amounts of FDs of both study groups.

Lateral cephalometric radiographs, which were considered the ‘gold standard’ in orthodontics in the past and are one of the clinical diagnostic tools, show the sagittal and vertical relationship of the jaw bones with respect to the skull base and enable a two-dimensional evaluation of the relationship of the anterior teeth with the jaw bones [42,43]. Korkmaz et al. [41] evaluated the changes in maxillary and mandibular bone structures after functional therapy with the Twinblock appliance using FD analysis on cephalometric and panoramic radiographs. They reported that there was a significant decrease in the FD values of the tuber maxilla, condyle, and corpus mandible of patients treated in the prepubertal period, and that there was no significant change in the FD values of patients treated in the post-pubertal period. In this presented study, MBG patients were selected from patients in the peak period and FFRDG patients were selected from patients in the post-peak period, in accordance with the indication of functional therapy. While a significant decrease was observed in buccoapical FD values of both study groups after functional therapy, changes in the symphysis region were not found to be significant.

It has been reported that areas of interest selected in larger sizes in FD analysis can provide more information about that region [44]. The limitations of this study are that the area of interest was selected at most 30 × 30 pixels in size in order to avoid including surrounding anatomical structures, and that adequate comparison could not be made since there are not many similar studies in the literature. In addition, it is thought that more studies are needed to examine the changes in alveolar bone thickness and height, especially in the buccoapical region. However, this presented study provides orthodontists with important insight into the clinical prognosis regarding the remodeling processes occurring in the mandibular anterior bone structures with functional therapy.

## 5. Conclusions

The first and second null hypotheses of this study were rejected. In light of this study, the following conclusions were reached:

- The effect of functional therapy on mandibular anterior bone structures in class II malocclusions can be evaluated using lateral cephalometric radiographs with FD analysis;
- It was observed that functional therapy caused buccoapical alveolar trabeculation changes and a decrease in the density of mandibular anterior bone structures, especially due to the forward movement of the lower incisors, compared to individuals who did not receive orthodontic treatment;
- Due to the significant negative correlation between the IMPA angle and the FD values of the alveolar bone in the buccoapical of the mandibular incisors, it was concluded that orthodontists should pay attention to the IMPA angle before functional therapy in order to prevent possible alveolar complications.

## Figures and Tables

**Table 1:** Patient data on age, gender, and therapy durations.

			CG	FFRDG	MBG
Age (year)	Mean ± SD		17.4 ± 3.1	14.6 ± 0.9	11.2 ± 1.2
Gender	Female	n-%	12–60%	9–45%	8–40%
	Male	n-%	8–40%	11–55%	12–60%
Therapy duration (month)				4.9 ± 1.1	10.1 ± 1.26

SD: standard deviation, n: sample. CG: control group, FFRDG: Forsus fatigue-resistant device group, MBG: Monoblock group.

**Table 2:** Statistical analysis results for intra- and inter-group comparisons of FD values.

			CG ^a^	FFRDG ^b^	MBG ^c^	*p*
Buccoapical	T0	Mean ± SD	1.745 ± 0.001	1.743 ± 0.001	1.746 ± 0.001	0.810 ^K^
	T1	Mean ± SD	1.745 ± 0.001	1.738 ± 0.011 ^a^	1.736 ± 0.012 ^a^	0.013 *^K^
	T0/T1 difference	Mean ± SD		0.007 ± 0.01	0.009 ± 0.01	0.841 ^M^
	*Intra-group difference p*			0.004 *^W^	0.004 *^W^	
Symphysis	T0	Mean ± SD	1.745 ± 0.001	1.744 ± 0.001	1.745 ± 0.001	0.592 ^K^
	T1	Mean ± SD	1.745 ± 0.001	1.743 ± 0.001	1.742 ± 0.01	0.815 ^K^
	T0/T1 difference	Mean ± SD		0.0001 ± 0.0004	0.002 ± 0.008	0.369 ^M^
	*Intra-group difference p*			0.863 ^W^	0.306 ^W^	

^K^: Kruskal–Wallis; ^M^: Mann–Whitney U test; ^W^: Wilcoxon test; *: *p* < 0.05; SD: standard deviation; CG: control group, FFRDG: Forsus fatigue-resistant device group, MBG: Monoblock group. ^a^: Difference with CG in the same row *p* < 0.05; ^b^: Difference with FFRDG in the same row *p* < 0.05; ^c^: Difference with MBG in the same row *p* < 0.05.

**Table 3:** Statistical analysis results for intra- and inter-group comparisons of IMPA.

		CG ^a^	FFRDG ^b^	MBG ^c^	*p*
T0	Mean ± SD	93.9 ± 3.8	90.8 ± 3.9 ^a^	90.3 ± 3.0 ^a^	0.005 *^A^
T1	Mean ± SD	93.9 ± 3.8	97.5 ± 4.0 ^a^	98.5 ± 4.5 ^a^	0.002 *^A^
T0/T1 difference	Mean ± SD		6.7 ± 1.3	8.2 ± 2.1	0.013 *^T^
*Intra-group difference p*			<0.001 *^P^	<0.001 *^P^	

^A^: One-way ANOVA; ^T^: Student’s *t* test; ^P^: Paired *t* test; *: *p* < 0.05; SD: standard deviation; CG: control group, FFRDG: Forsus fatigue-resistant device group, MBG: Monoblock group; ^a^: difference with CG in the same row *p* < 0.05; ^b^: difference with FFRDG in the same row *p* < 0.05; ^c^: difference with MBG in the same row *p* < 0.05.

**Table 4:** Statistical results regarding the correlation between IMPA and FD values in groups.

			IMPA	Buccoapical	Symphysis
For FFRDG	IMPA	Spearman rho	1	−0.459	−0.113
		*p*		0.042 *	0.634
	Buccoapical	Spearman rho		1	0.342
		*p*			0.14
	Symphysis	Spearman rho			1
For MBG	IMPA	Spearman rho	1	−0.510	−0.226
		*p*		0.022 *	0.337
	Buccoapical	Spearman rho		1	−0.07
		*p*			0.77
	Symphysis	Spearman rho			1

FFRDG: Forsus fatigue-resistant device group, MBG: Monoblock group, *: *p* < 0.05.

## Data Availability

All data supporting the results of this study are included within the article.
